# Does Metabolic Syndrome and Its Components Have Prognostic Significance for Renal and Cardiovascular Outcomes in IgA Nephropathy?

**DOI:** 10.3390/biomedicines12061250

**Published:** 2024-06-04

**Authors:** Balázs Sági, Tibor Vas, Botond Csiky, Judit Nagy, Tibor József Kovács

**Affiliations:** 1 Medical School, Clinical Center, 2nd Department of Internal Medicine and Nephrology, Diabetes Center, University of Pécs, 7624 Pécs, Hungary; balazs.sagidr28@gmail.com (B.S.); vas.tibor@pte.hu (T.V.); botond.csiky@gmail.com (B.C.); judit.nagy@aok.pte.hu (J.N.); 2 Triton Life Dialysis Center, 7624 Pécs, Hungary

**Keywords:** chronic kidney disease, IgA nephropathy, renal function, metabolic syndrome, arterial stiffness

## Abstract

Background: Patients with IgA nephropathy (IgAN), a chronic kidney disease (CKD), are significantly more likely to have cardiovascular (CV) mortality and morbidity than the general population. The occurrence of metabolic syndrome (MetS) and metabolic risk factors are independent risk factors for CV disease and renal progression. The purpose of this study was to determine how metabolic characteristics in a homogeneous population of CKD patients relate to prognosis. Methods: A total of 145 patients with CKD stages 1–4 diagnosed with IgA nephropathy (92 men and 53 women, aged 54.7 ± 13 years) were examined and monitored for a median of 190 months. All-cause mortality and any CV event, such as stroke, myocardial infarction, revascularization (CV), end-stage renal disease, and renal replacement therapy (renal), have been included in the composite endpoints (CV and renal). Results: Patients with MetS had significantly more primary endpoint events (23/65 patients vs. 15/60 patients, *p* < 0.001) compared to the non-MetS group. The MetS group had a statistically significant increase in both primary renal and CV endpoints (18/65 vs. 10/60, *p* = 0.001), and in CV endpoint events (7/65 vs. 6/60, *p* = 0.029) among the secondary endpoints (CV and renal separately). Based on Cox regression analysis, the main endpoint independent predictors of survival were dyslipidemia, eGFR, hemoglobin, urine albuminuria, and diabetes mellitus. Independent predictors of secondary renal endpoints were dyslipidemia, hemoglobin, urine albumin, and eGFR. Predictors of secondary cardiovascular endpoints were gender, BMI, and diabetes. When Kaplan–Meier curves were analyzed at the combined endpoints (CV and renal) or each endpoint independently, significant differences were seen between MetS and non-MetS. With more MetS components, the primary endpoint rate increased significantly (MetS comp. 0 vs. MetS comp. 2+, primary endpoints, *p* = 0.012). Conclusions: Our results show that the metabolic profile has a prognostic role not only for renal endpoints but also for CV endpoints in IgAN. BMI, hyperuricemia, hypertension, and diabetes have a predictive value for the prognosis of IgA nephropathy.

## 1. Introduction

Cardiovascular (CV) morbidity and mortality in patients with chronic kidney disease (CKD) is many times higher than in the general population [1,2], and in primary glomerular diseases such as IgA nephropathy (IgAN), the absolute risk of cardiovascular disease (CVD) is roughly 2.5 times higher than in the population without any kidney diseases [3]. The classification of CVD risk in these individuals may be improved by taking into account eGFR, proteinuria, the type of glomerular disease [3], and the patient’s metabolic profile. Moderate and severe CKDs are independent risk factors for cardiovascular disease [4]. IgA nephropathy is the most common primary glomerular disease worldwide and is a relatively common cause of end-stage kidney disease (ESKD) [5,6]. Long-term observational studies have shown that IgAN causes ESKD in 40% of patients within 20 years after diagnosis [7,8]. The clinical presentation is usually nephritic syndrome and hematuria with variable degrees of proteinuria. Pathologically, IgAN is defined by polymeric IgA1 being deposited in the glomerulus, primarily in the mesangium; mesangial hypercellularity and mesangial matrix expansion are also present, along with various degrees of interstitial fibrosis and glomerulosclerosis. The degree of glomerulosclerosis and tubulointerstitial fibrosis in renal pathology, the presence of hypertension and excessive proteinuria, and a marked decline in glomerular filtration rate (GFR) at the time of renal biopsy are all considered adverse prognostic indications [9,10].

Although research on MetS’s independent relationship with the onset and progression of CKD has been increasingly studied, the results are inconsistent. According to a population-based study, patients with non-diabetic early-stage CKD (stages 1–3) are the only ones whose MetS has a substantial effect on the progression of CKD; patients with non-diabetic late-stage and diabetic CKD are not affected [11]. Independent of diabetes grades, a different investigation found a substantial correlation between CKD and components of MetS [12]. Furthermore, despite a positive and considerable risk of estimated glomerular filtration rate (eGFR) decline, a previous cohort study found no correlation between MetS and incident CKD [13]. Several meta-analyses have been conducted to settle these disputes. According to a prior meta-analysis involving 11 cohort studies, MetS may promote the onset of CKD [14].

MetS and its components are putative risk factors for albuminuria and proteinuria, according to a different meta-analysis of 57 studies [15]. Previous meta-analyses, however, have solely comprised research on individuals without CKD. There have not been any meta-analyses conducted to look into the relationship between ESKD and renal events like eGFR decline and MetS and its components.

Apart from the aforementioned established risk factors, new research has also linked the advancement in the progression of IgAN to other cardiovascular risk factors, including hypertriglyceridemia, hyperuricemia, excessive body weight, and smoking [16,17]. However, there are limited data about the prevalence of metabolic syndrome and the metabolic profile association between metabolic syndrome (MetS) and renal and cardiovascular (CV) outcomes in IgAN patients. More focus is being placed on identifying and treating the modifiable risk factors that can stop or slow down the deterioration of renal function.

Earlier cross-sectional studies have also demonstrated a link between MetS and CKD [18,19]. Kurella et al. conducted only one longitudinal investigation [20], demonstrating a significantly increased risk of incident CKD in non-diabetic adults with MetS. However, in our study, we investigated the effects of MetS and metabolic profiles on the outcomes of IgAN. We explored metabolic syndrome components (hypertension, diabetes, obesity, dyslipidemia, hyperuricemia) separately and whether they have an impact on renal and CV outcomes.

## 2. Materials and Methods

**Figure 1:**
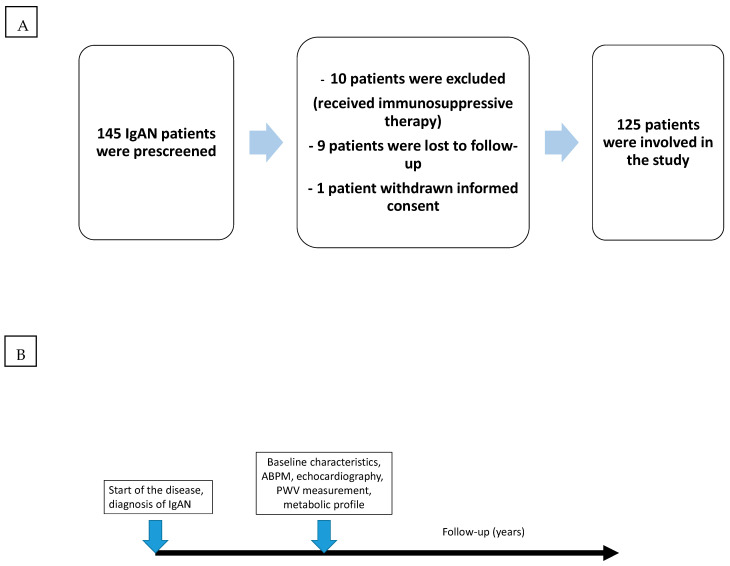
A flow chart of recruited patients (**A**), and a flow chart of the present study’s process (**B**).

We included 145 renal biopsy-proven IgAN patients in our follow-up study to our earlier 2013 investigation, and we analyzed their data prospectively (Figure 1A,B). The study procedure was approved by the University of Pécs Regional Research Ethics Committee, and all participants provided written consent for its completion.

The inclusion criteria for this study were confirmed IgAN and being over the age of 18. The exclusion criteria were previous or current immunosuppressive treatment (due to the modifying effects of the metabolic components) and severe comorbidities (such as malignancies that required active treatment or acute infection).

Metabolic syndrome (MetS) was defined as the NCEP ATP III (National Cholesterol Education Program Adult Treatment Panel III) criterion, which necessitates any three of the following five criteria: waist circumference (>102 cm in males, >90 cm in females) or BMI > 27 kg/m^2^, fasting glucose ≥ 5.6 mmol/L, triglyceride ≥ 1.7 mmol/L, HDL cholesterol <1.0 mmol/L in males and <1.3 in females, and systolic/diastolic blood pressure >130/85 mmHg.

An echocardiography examination was performed at the beginning of patient enrollment, and information about classic CV risk factors such as smoking, lipid abnormalities, obesity, diabetes, hypertension, and antihypertensive medications (ACEI/ARB, BB, and CCB) was also recorded. The obesity inclusion criterion was a BMI over 27 kg/m^2^. The CKD-EPI formula was used to estimate renal function (eGFR, mL/min, 1.73 m^2^). A 24 h blood pressure monitor was used, provided by ABPM (Meditech, Budapest, Hungary) devices, to determine the patient’s 24 h average systolic and diastolic blood pressure, pulse pressure, and diurnal index. At the start of the study, arterial stiffness and pulse wave velocity (PWV) were measured by a SphygmoCor System (AtCor Medical, Sydney, Australia). During these visits, medical events that had occurred since the previous visit were recorded, the patient’s physical status was examined, and detailed laboratory tests were performed. Blood pressure values were determined from the average of three measurements taken after 10 min of rest. Serum parameters were determined by implementing routine, standardized laboratory methods.

### 2.1. Endpoint Definition

The primary composite endpoints were all-cause mortality and any CV event, such as stroke, myocardial infarction, or revascularization. Secondary cardiovascular (CV) endpoints were any CV event, such as stroke, myocardial infarction, or any revascularization. Secondary renal endpoints were reaching end-stage renal disease and starting renal replacement therapy. We collected these data prospectively. 

### 2.2. Statistical Analysis

Statistical analyses were performed using SPSS 21.0 software (SPSS, Inc., Chicago, IL, USA). A comparison of clinical and laboratory parameters was made using Student’s *t*-test and ANOVA, as appropriate. The mean SD was used to express data from a normal distribution. Correlations between continuous variables were evaluated with linear regression using the Pearson test, and for categorical variables, the Spearman correlation test was used. The survival rates of the two groups were examined by using the Mantel-Cox log-rank test. The effect of metabolic syndrome components on survival was evaluated by using Cox regression analysis. Values of *p* < 0.05 were considered statistically significant.

## 3. Results

This study included 145 patients, but 20 patients were excluded: 10 patients due to immunosuppressive therapy, 9 patients lost before follow-up, and 1 patient who withdrew informed consent; so, in total, 125 patients’ data were investigated. The average age was 53.0 ± 12.7 years, and 92 were male. The median follow-up time was 190 ± 170 months. Patients were divided into two groups based on MetS presence or absence. Baseline clinical data are shown in Table 1. There were significant differences between the two groups in systolic blood pressure, pulse pressure, systolic diurnal rhythm, diastolic dysfunction, BMI, pre-diabetes (impaired fasting glucose: IFG; impaired glucose tolerance: IGT), eGFR, PWV, HDL cholesterol, TG, and uric acid, but not in age or gender. The distribution of metabolic parameters in the MetS presence and MetS absence groups is summarized in Table 2.

Primary combined endpoint and secondary renal and CV endpoint event rates are presented in Table 3 in cases of the presence of MetS components and MetS combined with PWV.

**Figure 2:**
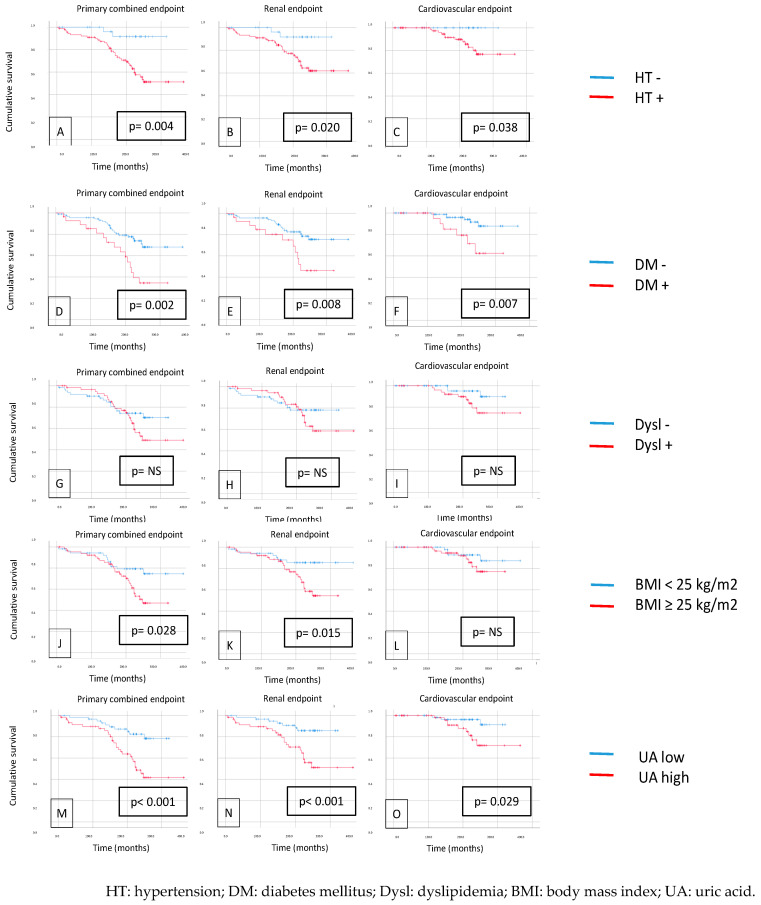
The Kaplan–Meier curves demonstrate the following primary endpoints: primary combined (**A**), renal (**B**), and cardiovascular (**C**) endpoints in patients with or without hypertension; primary combined (**D**), renal (**E**), and cardiovascular (**F**) endpoints in patients with diabetes; primary combined (**G**), renal (**H**), and cardiovascular (**I**) endpoints in patients with dyslipidemia; primary combined (**J**), renal (**K**), and cardiovascular (**L**) endpoints in patients with a BMI of 25 kg/m^2^; primary combined (**M**), renal (**N**), and cardiovascular (**O**) endpoints in patients with and without hyperuricemia.

When we investigated MetS components (HT, DM, BMI, dyslipidemia), the K-M curves showed significantly worse survival in cases of hypertension (primary endpoint: *p* = 0.004, secondary renal endpoint: *p* = 0.020, secondary CV endpoint: *p* = 0.0038), diabetes mellitus (*p* = 0.002, *p* = 0.008, *p* = 0.007), and a higher BMI (cut-off 25 kg/m^2^) (*p* = 0.028, *p* = 0.015, *p* = NS) in both primary and secondary endpoints. Surprisingly, there was a non-significant difference in the primary and secondary outcomes in the case of dyslipidemia, but we found significant differences in the case of hyperuricemia (cut-off 360 µmol/L) (*p* < 0.001, *p* < 0.001, *p* = 0.029) (see Figure 2).

**Figure 3:**
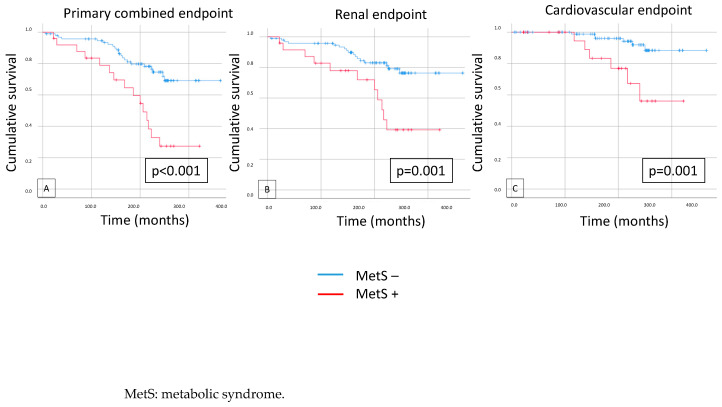
Kaplan–Meier curves show primary combined (**A**), renal (**B**), and cardiovascular (**C**) endpoints in cases of patients with and without metabolic syndrome.

There were significant differences in survival on the K-M curves in the case of the primary combined endpoint (*p* < 0.001) and the secondary renal (*p* = 0.001) and cardiovascular endpoints (*p* = 0.001) when we compared the IgAN patients with and without MetS (Figure 3).

**Figure 4:**
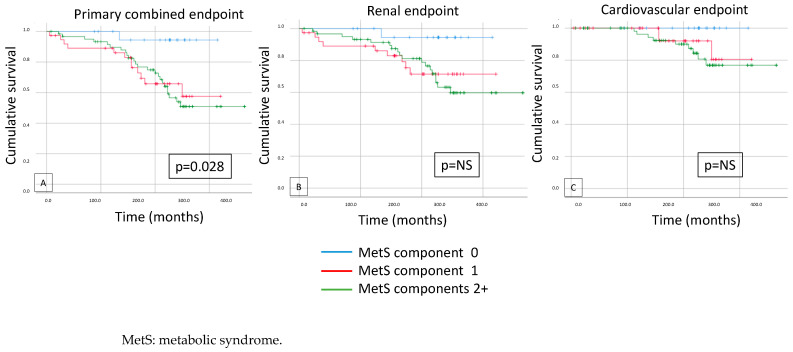
Assuming metabolic syndrome components are detected, the Kaplan–Meier curves show the primary combined (**A**), renal (**B**), and cardiovascular (**C**) outcomes (0 vs. 1 vs. 2+).

There was a significant difference in survival only in the primary endpoint when we compared the patients with MetS components (0 vs. 1. vs. 2+); the secondary endpoints were not significantly different (*p* = 0.028) (Figure 4).

**Figure 5:**
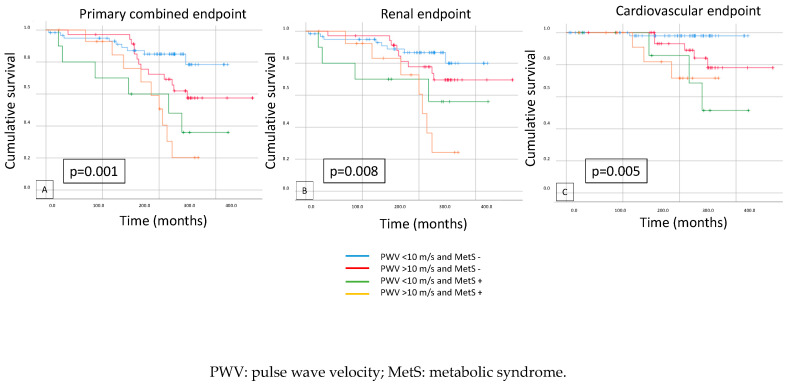
Kaplan–Meier curves show primary combined (**A**), renal (**B**), and cardiovascular (**C**) endpoints in cases of low or high PWV (<10 m/s vs. ≥10 m/s) and metabolic syndrome presence or absence (Met sy − vs. Met sy +).

When we combined arterial stiffness, PWV (<10 m/s vs. ≥10 m/s), and MetS presence or absence in the survival examination, there was a significant difference between the low-PWV group without MetS compared to the high-PWV group with MetS. All endpoints, the primary combined endpoint (*p* = 0.001) and both secondary endpoints (renal and cardiovascular) (*p* = 0.008; *p* = 0.005), were significant (Figure 5).

Multivariate analyses were performed on the clinical data, metabolic parameters, echocardiographic parameters, and laboratory results. The significant factors in the competitive risk model were gender (OR = 4.333, *p* = 0.001), age (OR = 2.906, *p* = 0.026), dyslipidemia (OR = 3.474, *p* = 0.034), hypertension (OR = 5.806, *p* = 0.018), diabetes (OR = 1.912, *p* = 0.011), BMI (OR = 2.205, *p* = 0.021), eGFR (OR = 3.187, *p* = 0.021), hemoglobin (OR = 2.237, *p* = 0.029), albuminuria (OR = 2.568, *p* = 0.013), and uric acid level (OR = 1.837, *p* = 0.021) (Table 4).

The primary endpoint independent predictors of survival were dyslipidemia, eGFR, hemoglobin, urine albuminuria, and diabetes mellitus, as determined by using Cox regression analysis. The secondary renal endpoint independent predictors were dyslipidemia, hemoglobin, urine albuminuria, and eGFR. The secondary cardiovascular endpoint predictors were gender, BMI, and diabetes (Table 5).

Cox regression analysis adjusted for MetS components showed that the primary endpoint and the secondary renal endpoint independent predictors were uric acid and diabetes (Table 6).

## 4. Discussion

In the present study, we demonstrated that metabolic syndrome and its components, especially hypertension, diabetes, obesity, and hyperuricemia, were significantly associated with the primary combined endpoint (renal plus CV) and the secondary renal and cardiovascular outcomes, but dyslipidemia was not, in a relatively small number of patients, which are counterbalanced by the long follow-up. Due to the variability in serum lipids, one measurement had no impact on CV or renal endpoints. MetS development with increased pulse wave velocity (PWV) showed a worse renal and CV prognosis.

Survival curves stratified by MetS status (at least three factors from the above-mentioned ones) showed significant differences in the association with the various endpoints in a longer follow-up.

The key objective of the MetS diagnosis criteria was to identify the population most at risk of developing cardiovascular diseases [21,22]. In today’s world, it is undeniable that individuals with MetS have a markedly increased risk of developing both CVD and CKD [14,23,24,25,26]. The majority of the observational studies covered in these articles discovered a strong correlation between MetS and CKD. Nevertheless, as most of the research was cross-sectional, it was unable to identify whether MetS is linked to long-term changes in renal function or to demonstrate a cause-and-effect relationship between the decline in kidney function and MetS.

In our earlier research, we found a strong correlation between MetS and the progressive loss of kidney function, except for ESKD, which may have an impact on the progression of the earliest stages of IgAN [23]. The present data support these observations, and not only the renal endpoints but also the CV endpoints are affected by the presence of MetS in IgAN.

Lee et al. found that MetS had an impact on CKD progression only in early-stage CKD patients in their short follow-up (average 10.6 months) study [11]. The short follow-up may be the reason that the impact of MetS was not observed among late-stage and diabetic CKD patients. In the present study, we had more than ten times longer follow-up, clearly demonstrating the effect of MetS on the primary combined (CV plus renal) and secondary renal endpoints, despite the lower number of cases.

The other important difference was that the proportion of diabetic patients was higher (in Lee et al.’s study, 53% vs. 24%), and our patients were homogenous for CKD.

The two main causes of CKD worldwide, diabetes and hypertension, are among the elements of MetS that have been extensively studied and reported. Regarding the connection between diabetes and IgAN, Fliser et al. [27] reported that patients with IgAN or ADPKD who have incipient chronic renal disease already exhibit insulin resistance and hyperinsulinemia. These early changes may be worsened by other MetS factors. 

At the start of the patient follow-up in our current investigation, 30 patients, or 24% of the total, had diabetes mellitus (see Table 1), which is nearly double the prevalence of DM in the whole population. It is recognized that in patients with diabetes, IgAN may also be the only renal abnormality or that it may superimpose on diabetic nephropathy. Because IgA1 immune complexes or aggregates can be more easily deposited in diabetic patients’ glomeruli due to metabolic changes, intraglomerular hypertension, and hyperfiltration, the relationship between IgAN and diabetes may not be a coincidence. Moreover, type 2 diabetes is frequently associated with malformations of the IgA immune system [28].

Common in CKD patients, hypertension is a predictor of cardiovascular morbidity and mortality, the same as it is in the general population. A U-shaped relationship has been shown between blood pressure and mortality. Early mortality is predicted not only by high blood pressure but also by low mean systolic and diastolic blood pressure [29]. The progression of IgAN is more severe when hypertension is present [30]. It was therefore not surprising that among the MetS parameters examined in our investigation, hypertension had the highest impact on the prognosis of IgAN, consistent with our previous recommendation that IgAN patients require strict blood pressure control [31].

Numerous epidemiological studies have linked obesity to an increased risk of developing CKD and ESKD [32,33,34]. Regardless of the existence of diabetes or hypertension, obesity, as measured by an elevated BMI, was linked to ESKD and reduced renal function in the populations under investigation. Losing weight slows the advancement of chronic kidney disease [35]. Obesity was found to be a predictive factor for both the development of ESKD and hypertension in IgAN patients. Obese patients also exhibit glomerular enlargement and ultrastructural modification of the glomerular basement membrane, which are well-documented phenomena [27,36,37]. The present study and our former observation are also able to confirm this [23]. 

Observational studies and meta-analyses have shown dyslipidemia, namely, atherogenic dyslipidemia (high triglyceride and low HDL cholesterol), to be an independent risk factor for the onset and course of chronic kidney disease (CKD) [38,39]. Dyslipidemia was an important, significant factor according to Cox regression analysis in the primary combined endpoints and secondary renal endpoints (see Table 3). The lower number of secondary CV endpoints may explain the non-significant connection between secondary CV endpoints and dyslipidemia. 

Moreover, the progressive deterioration of renal function may increase oxidative stress and inflammation, which may trigger several metabolic changes, including hypertriglyceridemia, diabetes, and insulin resistance. These changes have the potential to create vicious cycles. In the study of Syrjänen et al. [16], elevated triglyceride levels were associated with progressive IgAN. Our long-term follow-up also supports these findings (see Table 3).

According to recent epidemiologic and experimental data, hyperuricemia may play a part in IgAN as well as serving as a risk factor for the onset and progression of renal illness and a marker of decreased kidney function [40,41].

In addition to being linked to renal tissue inflammation in patients with IgAN, serum uric acid may also have another role in the development of tubulointerstitial lesions [42]. In the Jerusalem Lipid Research Clinic cohort trial, serum uric acid was a long-term predictor of acute kidney injury and CKD. This was not dependent on GFR [43], based on a follow-up of 2449 patients. Our results were very similar in a homogenous but lower number of patient groups with long follow-ups. 

Previous meta-analyses have indicated that individuals with MetS have a higher chance of developing chronic kidney disease (CKD), as evidenced by albuminuria or proteinuria [15] and a reduction in eGFR [14,44]. The results of previous research that examined the role of MetS and its components in the risk of developing renal disease in addition to incident CKD are inconsistent. The significant role that MetS plays in the evolution of CKD was highlighted by a large prospective cohort study conducted across the United States. This demonstrated that people with MetS had a two-fold increased risk of developing ESKD compared to people without the condition [45]. 

On the other hand, an African American study’s secondary analysis showed that MetS is not directly linked to the advancement of CKD. The relationship between it and CKD acceleration is complicated by other variables [46].

Li et al.’s meta-analysis provided evidence for a strong correlation between MetS and the acceleration of renal failure, indicating that MetS may be a separate predictor of disease progression from acute CKD [47]. In both industrialized and developing nations, diabetes and hypertension are the main causes of both CKD and ESKD [48]. However, their study indicated that the risk was highest for increased blood pressure and lowest for IFG. Similar results were observed among our IgAN patients (see Table 5). A few investigations have confirmed the link between increased blood pressure and renal insufficiency [49,50]. The present study’s risk estimate is consistent with earlier meta-analyses [44], which found a positive correlation between high blood pressure and poor kidney function, with an odds ratio (RR) of 1.37 (1.29–1.46). 

Conversely, prior research examining the correlation between glycemic status and renal disease has produced contradictory results. According to a recent meta-analysis, the risk of CKD was found to be slightly enhanced by pre-diabetes (RR = 1.11, 95% CI 1.02–1.21), which includes IFG (IGT), and elevated glycated hemoglobin A1c [51]. In the non-diabetic group, IFG was not causally linked to the development of CKD, according to a Mendelian randomization study [52]. This difference may be due to the unique characteristics of the study participants. Individuals with diabetes at baseline or during follow-up may have been included in studies addressing IFG as part of MetS, whereas individuals with IGT may not have been included. Therefore, depending on different glycemic exposures, the link between IFG and renal impairment may change. Therefore, it is important to consider MetS when interpreting the risk estimations. IFG is not usually reported by itself; rather, it is thought to be the outcome of intricate interactions between several MetS components [53], especially in individuals with chronic kidney disease (CKD) who are more likely to have prior cardiovascular disease, diabetes, hypertension, and abnormal lipid metabolism [54,55]. This may also explain the inconsistency in our results.

In an individual-level meta-analysis of 5.5 million people in 39 general population cohorts, BMI values of 30, 35, and 40 kg/m^2^ were associated with an elevated risk of eGFR drop ≥40% by 18%, 69%, and 102%, respectively [34]. When comparing patients with and without baseline CKD, the relationship was quite similar.

Studies on dyslipidemia and renal dysfunction have looked at different phases of chronic kidney disease. A cohort study found that among individuals without chronic kidney disease (CKD), increased TG was a risk factor contributing to the observed link between MetS and deterioration in renal function [56]. The data from a different investigation revealed that while high TG levels had no or inverse relationships with time to end-stage renal disease (ESKD) in CKD stages 4–5, they were linked to a higher incidence of CKD and a faster deterioration in renal function in non-CKD and CKD stage 3 [57]. In a different study, there was also no significant link found between TG and HDL cholesterol levels and the development of renal replacement therapy and rapid renal progression in CKD stages 3–5 [58]. 

The consequences of hyperlipidemia in patients with advanced chronic kidney disease (CKD), especially those with end-stage renal disease (ESKD), may be obscured by the presence of more potent conventional CVD risk factors. 

Our results allow nephrologists to concentrate on the initial phases of chronic kidney disease (CKD), perhaps resulting in the early implementation of therapies for metabolic disorders, like dietary changes or medication. Furthermore, prior research has shown that the distribution of fat, namely, the amount in the abdomen rather than overall obesity, is a significant factor in poor renal outcomes [59]. However, the current study did not perform a subgroup analysis based on fat distribution. There was a significantly higher abdominal circumference in males (*p* = 0.030) and females (*p* = 0.023) in the MetS group compared to the non-MetS group.

Lin et al. demonstrated that blood pressure is the most important component of MetS for renal outcomes in CKD 1–4 stage patients. The MetS group had a higher risk for renal outcomes and all-cause mortality (HR: 1.62 and 1.43) [60], and our results also confirm this (see Table 4).

We demonstrated in our previous study that arterial stiffness had predictive value for renal and CV prognosis in autosomal polycystic kidney disease [61], and the etiology of the kidney disease did not similarly alter the vascular function of different CKD groups. Blood pressure was an independent risk factor for PWV [62]. Arterial stiffness was associated with GFR and left ventricular hypertrophy (LVH) in IgAN [63]. In this study, we could demonstrate that if we combined MetS with an arterial stiffness parameter (PWV), the renal and cardiovascular risk prediction would be more pronounced. Vascular stiffness measurements could help nephrologists with further risk assessment.

In our other earlier study, we found an association between LVH and eGFR in IgAN. There was a strong correlation between the left ventricular mass index (LVMI) and renal function in a homogenous immunocomplex-mediated CKD population of IgAN patients. An independent predictor of the LVMI was the initial stage of renal function. Renal composite endpoints and CV were higher when the LVMI was higher. An independent predictor of ESKD and CV events may be a higher LVMI. An increased LVMI should draw attention to CKD patients who require closer monitoring, referral for additional CV testing, and maximal renal and heart protection since they have a higher risk of cardiovascular and renal disease at an early stage of the disease (II–IV) [64]. In our current study, there was no significant difference in the LVMI between the MetS and non-MetS groups. 

## 5. Limitations of this Study

Firstly, this study has a relatively small sample size and a small endpoint event rate, but this is a medium-term follow-up study conducted at a single center. Secondly, this cohort provides the basis for the cut-off value and the single baseline measurement. It might not be suitable for other racial and ethnic groups as a result. Thirdly, residual confounding effects from lifting style, drugs that alter CV risk, and comorbidity might introduce bias into prospective analyses. For this reason, larger sample sizes and more prospective research are required. Fourth, there were more men than women in this study, which could have influenced the findings.

## 6. Conclusions

In conclusion, our study proved that despite the lower number of cases, but longer prospective follow-up, the importance of metabolic changes at the start of IgAN have an important impact on the outcome. Therefore, risk stratification of IgAN patients is very helpful in identifying high-risk individuals, especially when the metabolic profile is combined with an increased arterial stiffness evaluation for additional risk. Further, multicenter, randomized clinical trials with a large number of patients are warranted to confirm our results.

Our results also confirm that complex metabolic risk reduction therapy is necessary in all IgAN patients, especially in high-risk groups, which contains ACEI/ARB, statins, uric acid-lowering agents in cases of lower GFR (<60 mL/min) and proteinuria, as well as today’s SGLT-2-inhibitors [65,66].

## Figures and Tables

**Table 1:** Baseline clinical data of IgAN patients.

Clinical Data (n = 125)	Met sy − (n = 60)	Met sy + (n = 65)	*p*
Man/woman (n/%)	36/24 (60/40)	46/19 (71/29)	0.079
Age (year) (mean and 25–75th percentiles)	53.2 (43.0–63.0)	55.4 (44.0–64.0)	0.109
Average systolic BP (Hgmm) (mean and 25–75th percentiles)	123.5 (115.0–129.25)	127.4 (117.3–131.2)	0.002 *
Average diastolic BP (Hgmm)	73 ± 9.6	75.7 ± 9.5	0.231
24 h pulse pressure (Hgmm) (mean and 25–75th percentiles)	49.35 (43.0–54.0)	53.10 (44.5–56.5)	0.012 *
Diurnal index systolic (%)	10.92 ± 5.08	8.36 ± 7.72	0.020 *
Abdominal circumference in males (cm)	100.2 ± 4.2	112.1 ± 6.5	0.030 *
Abdominal circumference in females (cm)	90.1 ± 5.7	94.4 ± 7.3	0.023 *
Metabolic Parameters			
Hypertension (n,%)	41 (68)	53 (81)	0.118
BMI (kg/m^2^) (mean and 25–75th percentiles)	26.5 (22.9–29.7)	28.6 (23.4–30.1)	0.001 *
Dyslipidemia (n,%)	24 (40)	34 (52)	0.137
Diabetes (n, %)	9 (15)	21 (32)	0.087
IFG and IGT (n/%)	2 (3)	10 (15)	0.025 *
Overweighted (n/%)	3 (5)	5 (8)	0.098
Obesity (n/%)	2 (3)	32 (49)	0.001 *
Visceral obesity (n/%)	2 (3)	28 (43)	0.001 *
eGFR (mL/min)	94.6 ± 29.3	78.9 ± 37.9	0.005 *
eGFR < 60 mL/min (n/%)	2 (3)	4 (6)	0.086
Duration of kidney disease (year)	10.2 ± 9.7	8.8 ± 9.1	0.101
Smoking (n, %)	7 (12)	11(17)	0.156
Therapy			
ACEI/ARB (n, %)	46 (77)	60 (92)	0.079
BB (n, %)	12 (20)	19 (29)	0.178
Statin (n, %)	16 (27)	22 (34)	0.164
CCB (n, %)	9 (15)	19 (29)	0.082
Echocardiographic Parameters			
LVEF (%) (mean and 25–75th percentiles)	62.8 (59.0–66.5)	63.5 (60.1–66.7)	0.211
LVMI (g/m^2^)	103.53 ± 15.95	109.21 ± 21.25	0.123
LVEDD (cm)	6.05 ± 6.29	5.57 ± 5.13	0.173
DD (n/%)	7 (11)	17 (26)	<0.001 *
E/A	1.18 ± 0.32	0.93 ± 0.30	<0.001 *
Arterial Stiffness			
cfPWV (m/s) (mean and 25–75th percentiles)	9.97 (8.48–11.35)	11.34 (10.1–12.2)	0.003 *
Laboratory Results			
Hb (g/dL)	13.9 ± 1.6	13.6 ± 1.7	0.245
AU (mg/day) (mean and 25–75th percentiles)	457.48 (65.0–700.0)	558.34 (75.1–789.1)	0.078
UA (µmol/L)	303 ± 97.4	342 ± 84.9	0.009 *
Total cholesterol (mmol/L) (mean and 25–75th percentiles)	4.97 (4.28–5.51)	4.79 (4.35–5.41)	0.124
HDL cholesterol (mmol/L) (mean and 25–75th percentiles)	1.27 (1.03–1.44)	1.21 (1.0–1.38)	0.029 *
TG (mmol/L) (mean and 25–75th percentiles)	1.72 (0.93–2.04)	1.99 (0.99–2.34)	0.012 *
Hypercholesterinemia (n/%)	9 (15)	21 (32)	0.04 *
Hypertriglyceridemia (n/%)	5 (8)	52 (80)	0.001 *
Earlier CV Disease			
Heart failure	0 (0)	1(1)	0.176
Stroke	0 (0)	1 (1)	0.187
CAD	1 (2)	3 (5)	0.087
COPD	0 (0)	1 (1)	0.139

* = *p* < 0.05. BP: blood pressure; BMI: body mass index; IFG: impaired fasting glucose; IGT: impaired glucose tolerance; eGFR: estimated glomerular filtration rate; ACEI: angiotensin-converting enzyme inhibitor; ARB: angiotensin receptor blocker; BB: beta blocker; CCB: calcium channel blocker; CAD: coronary artery disease; LVEF: left ventricle ejection fraction; LVMI: left ventricle mass index; LVEDD: left ventricular end-diastolic diameter; DD: diastolic dysfunction; E/A: early and late mitral inflow; cfPWV: carotid–femoral pulse wave velocity; Hb: hemoglobin; AU: albuminuria; UA: uric acid; HDL cholesterol: high-density lipoprotein cholesterol; TG: triglyceride; CAD: coronary artery disease; COPD: chronic pulmonary obstructive disease.

**Table 2:** Distribution of metabolic parameters in MetS − and MetS + groups.

	HT (n/%)	IFG/ IGT (n/%)	DM (n/%)	Obesity (n/%)	Triglyceride (n/%)	HDL Cholesterol (n/%)	Number of Positive Parameters/ Patients (average/n)
MetS + (n = 65)	53 (82)	10 (15)	21 (32)	32 (49)	52 (80)	33 (51)	201 (3.09)
MetS − (n = 60)	41 (68)	2 (3)	9 (15)	2 (3)	5 (8)	15(25)	74 (1.23)

HT: hypertension; IFG: impaired fasting glucose; IGT: impaired glucose tolerance; DM: diabetes mellitus; MetS: metabolic syndrome; HDL: high-density lipoprotein.

**Table 3:** Primary combined endpoint and secondary renal and CV endpoint event rates.

Parameters	Primary Combined Endpoint Rate (n/%)	Secondary Renal Endpoint Rate (n/%)	Secondary CV Endpoint Rate (n/%)
HT− (n = 30)	2 (7)	2 (7)	0 (0)
HT+ (n = 95)	36 (38)	26 (27)	13 (14)
DM− (n = 95)	22 (23)	17 (18)	6 (6)
DM+ (n = 30)	16 (53)	11 (37)	7 (23)
Dyslipidemia− (n = 67)	16 (24)	12 (18)	4 (6)
Dyslipidemia+ (n = 58)	22 (38)	16 (27)	9 (15)
BMI low (n = 57)	11 (19)	7 (12)	4 (7)
BMI high (n = 68)	27 (40)	21 (31)	9 (13)
UA low (n = 65)	11 (17)	7 (11)	4 (6)
UA high (n = 60)	27 (45)	21 (35)	9 (15)
MetS− (n = 60)	15 (25)	10 (17)	6 (10)
MetS+ (n = 65)	23 (35)	18 (27)	7 (11)
MetS component 0 (n = 35)	1 (3)	1 (3)	0 (0)
MetS component 1 (n = 22)	8 (36)	9 (41)	3 (13)
MetS components 2+ (n = 68)	29 (42)	18 (26)	10 (15)
PWV < 10 m/s and MetS− (n = 61)	9 (15)	8 (13)	1 (2)
PWV > 10 m/s and MetS+ (n = 38)	13 (34)	9 (23)	5 (13)
PWV < 10 m/s and MetS− (n = 10)	6 (60)	4 (40)	3 (30)
PWV > 10 m/s and MetS+ (n = 16)	10 (62)	7 (44)	4 (25)

HT: hypertension; DM: diabetes mellitus; BMI: body mass index; UA: uric acid; MetS: metabolic syndrome; PWV: pulse valve velocity.

**Table 4:** Multivariate analyses on clinical data, metabolic parameters, echocardiographic parameters, and laboratory results in competitive risk model.

Clinical Data (n = 125)	OR	CI (95%)	*p*
Gender	4.333	3.973–4.761	0.001 *
Age	2.906	2.198–3.214	0.026 *
Average systolic BP	0.800	0.290–0.993	0.354
Average diastolic BP	0.576	0.119–0.626	0.615
24 h pulse pressure	0.737	0.174–0.947	0.535
Diurnal index systolic	0.559	0.283–0.874	0.693
Metabolic Parameters			
HT	5.806	5.301–6.455	0.018 *
DM	1.912	1.808–2.178	0.011 *
BMI	2.205	1.913–2.742	0.021 *
Dyslipidemia	3.474	2.237–4.546	0.034 *
IFG and IGT	0.564	0.118–0.922	0.787
Overweighted	0.479	0.340–0.941	0.109
Obesity	0.367	0.204–0.530	0.607
eGFR	3.187	2.455–4.366	0.021 *
Duration of kidney disease	0.718	0.387–0.972	0.284
Smoking	0.341	0.327–0.823	0.499
Echocardiographic Parameters			
LVEF	0.635	0.602–0.968	0.526
LVMI	0.460	0.068–0.691	0.772
LVEDD	0.508	0.285–0.952	0.293
Laboratory Results			
Hb	2.237	2.151–2.486	0.029 *
AU	2.568	1.933–3.653	0.013 *
UA	1.837	1.735–1.952	0.021 *
Total cholesterol	0.903	0.450–0.937	0604
HDL cholesterol	0.476	0.045–0.846	0.997
TG	0.806	0.463–0.944	0.143

* = *p* < 0.05. BP: blood pressure; HT: hypertension; DM: diabetes mellitus; BMI: body mass index; IFG: impaired fasting glucose; IGT: impaired glucose tolerance; eGFR: estimated glomerular filtration rate; LVEF: left ventricle ejection fraction; LVMI: left ventricle mass index; LVEDD: left ventricular end-diastolic diameter; Hb: hemoglobin; AU: albuminuria; UA: uric acid; HDL cholesterol: high-density lipoprotein cholesterol; TG: triglyceride.

**Table 5:** Cox regression for primary and secondary renal and cardiovascular endpoints.

	B	*p*	Exp(B)	95% CI for Exp(B) Lower	95% CI for Exp(B) Upper
Primary combined endpoint					
Gender	−0.898	0.078	0.408	0.150	1.104
Age	0.027	0.093	1.028	0.995	1.061
Dyslipidemia	1.144	0.034 *	3.140	1.091	9.042
HT	−0.774	0.363	0.461	0.087	2.447
DM	−0.964	0.031 *	0.381	0.159	0.914
BMI	0.014	0.787	1.014	0.916	1.123
eGFR	−0.021	0.010 *	0.980	0.964	0.995
Hb	−0.344	0.006 *	0.709	0.555	0.905
AU	0.001	0.001 *	1.001	1.001	1.002
UA	0.004	0.083	1.004	0.999	1.009
Secondary renal endpoint					
Gender	−0.492	0.416	0.611	0.186	2.003
Age	0.021	0.234	1.021	0.986	1.058
Dyslipidemia	1.964	0.003 *	7.130	1.931	26.328
HT	−0.743	0.430	0.476	0.075	3.011
DM	−0.568	0.285	0.567	0.200	1.605
BMI	0.087	0.151	1.091	0.969	1.228
eGFR	−0.030	0.004 *	0.971	0.951	0.991
Hb	−0.493	0.002 *	0.611	0.444	0.841
AU	0.002	0.001 *	1.002	1.001	1.002
UA	0.005	0.119	1.005	0.999	1.011
Secondary CV endpoint					
Gender	−2.632	0.029 *	0.072	0.007	0.759
Age	0.072	0.095	1.075	0.987	1.170
Dyslipidemia	0.571	0.531	1.771	0.296	10.581
HT	−11.318	0.961	0.001	0.001	126.263
DM	−2.240	0.002 *	0.106	0.025	0.454
BMI	−0.231	0.029 *	0.794	0.646	0.976
eGFR	−0.002	0.874	0.998	0.969	1.027
Hb	−0.260	0.192	0.771	0.521	1.140
AU	0.001	0.744	1.000	0.999	1.001
UA	0.002	0.542	1.002	0.995	1.010

* = *p* < 0.05. HT: hypertension; DM: diabetes mellitus; BMI: body mass index; eGFR: estimated glomerular filtration rate; Hb: hemoglobin; AU: albuminuria; UA: uric acid.

**Table 6:** Cox proportional hazard ratios of the metabolic syndrome parameters for the primary and secondary endpoints (adjusted for metabolic syndrome components).

	B	*p*	Exp(B)	95% CI for Exp(B) Lower	95% CI for Exp(B) Upper
Primary endpoint					
Dyslipidemia	−0.008	0.981	0.992	0.493	1.995
HT	−1.249	0.102	0.287	0.064	1.279
DM	−0.800	0.051	0.449	0.201	1.002
BMI	−0.013	0.743	0.987	0.913	1.067
UA	0.006	0.002 *	1.006	1.002	1.009
Secondary renal endpoint					
Dyslipidemia	0.114	0.777	1.121	0.508	2.475
HT	−0.828	0.290	0.437	0.094	2.024
DM	−0.549	0.258	0.578	0.223	1.496
BMI	0.040	0.357	1.041	0.955	1.135
UA	0.006	0.003 *	1.006	1.002	1.010
Secondary CV endpoint					
Dyslipidemia	−0.728	0.254	0.483	0.138	1.687
HT	−12.342	0.971	0.001	0.001	8.049
DM	−1.840	0.005 *	0.159	0.044	0.567
BMI	−0.128	0.077	0.880	0.763	1.014
UA	0.004	0.150	1.004	0.998	1.010

* = *p* < 0.05. HT: hypertension, DM: diabetes mellitus, BMI: body mass index, and UA: uric acid.

## Data Availability

The data underlying this article cannot be shared publicly due to Hungarian regulations and the privacy of individuals who participated in this study. The data could be shared upon reasonable request to the corresponding author if accepted by the Regional Committee for Medical and Health Research Ethics and local data protection officials.

## Abbreviations

ACEI/ARB	angiotensin-converting enzyme inhibitor/angiotensin receptor blocker
ADPKD	autosomal dominant polycystic kidney disease
AU	albuminuria
BB	beta blocker
BMI	body mass index
BP	blood pressure
CAD	coronary artery disease
CCB	calcium channel blocker
CKD	chronic kidney disease
COPD	chronic pulmonary obstructive disease
CV	cardiovascular
CVD	cardiovascular disease
DD	diastolic dysfunction
DM	diabetes mellitus
E/A	early and late mitral inflow
eGFR	estimated glomerular filtration rate
ESKD	end-stage kidney disease
Hb	hemoglobin
HDL	high-density lipoprotein
HT	hypertension
IFG	impaired fasting glucose
IgAN	immunoglobulin-A nephropathy
IGT	impaired glucose tolerance
LVEF	left ventricle ejection fraction
LVEDD	left ventricular end-diastolic diameter
LVH	left ventricular hypertrophy
LVMI	left ventricular mass index
MetS	metabolic syndrome
NCEP	ATP III National Cholesterol Education Program Adult Treatment Panel III
cfPWV	carotid–femoral pulse wave velocity
SGLT-2-inhibitor	sodium–glucose transporter-2-inhibitor
TG	triglyceride
UA	uric acid
